# Efficacy of Single-Dose Primaquine With Artemisinin Combination Therapy on Plasmodium *falciparum* Gametocytes and Transmission: An Individual Patient Meta-Analysis

**DOI:** 10.1093/infdis/jiaa498

**Published:** 2020-08-11

**Authors:** Kasia Stepniewska, Georgina S Humphreys, Bronner P Gonçalves, Elaine Craig, Roly Gosling, Philippe J Guerin, Ric N Price, Karen I Barnes, Jaishree Raman, Menno R Smit, Umberto D’Alessandro, Will J R Stone, Anders Bjorkman, Aaron M Samuels, Maria I Arroyo-Arroyo, Guido J H Bastiaens, Joelle M Brown, Alassane Dicko, Badria B El-Sayed, Salah-Eldin G Elzaki, Alice C Eziefula, Simon Kariuki, Titus K Kwambai, Amanda E Maestre, Andreas Martensson, Dominic Mosha, Richard O Mwaiswelo, Billy E Ngasala, Joseph Okebe, Michelle E Roh, Patrick Sawa, Alfred B Tiono, Ingrid Chen, Chris J Drakeley, Teun Bousema

**Affiliations:** 1 WorldWide Antimalarial Resistance Network, Oxford, United Kingdom; 2 Centre for Tropical Medicine and Global Health, Nuffield Department of Clinical Medicine, University of Oxford, Oxford, United Kingdom; 3 Infectious Diseases Data Observatory, Oxford, United Kingdom; 4 Green Templeton College, University of Oxford, Oxford, United Kingdom; 5 Department of Immunology and Infection, London School of Hygiene and Tropical Medicine, London, United Kingdom; 6 Department of Epidemiology and Biostatistics, University of California San Francisco, San Francisco, California, USA; 7 Global Health Group, Malaria Elimination Initiative, University of California, San Francisco, California, USA; 8 Global and Tropical Health Division, Menzies School of Health Research and Charles Darwin University, Darwin, Norther Territory, Australia; 9 Mahidol-Oxford Tropical Medicine Research Unit, Faculty of Tropical Medicine, Mahidol University, Bangkok, Thailand; 10 University of Cape Town/Medical Research Council Collaborating Centre for Optimising Antimalarial Therapy, University of Cape Town, Cape Town, South Africa; 11 Division of Clinical Pharmacology, Department of Medicine, University of Cape Town, Cape Town, South Africa; 12 Centre for Emerging Zoonotic and Parasitic Diseases, National Institute for Communicable Diseases, National Health Laboratory Services, Johannesburg, South Africa; 13 Wits Research Institute for Malaria, Faculty of Health Sciences, University of Witwatersrand, Johannesburg, South Africa; 14 Department of Clinical Sciences, Liverpool School of Tropical Medicine, Liverpool, United Kingdom; 15 Medical Research Council Unit The Gambia, London School of Hygiene and Tropical Medicine, London, United Kingdom; 16 Department of Medical Microbiology, Radboud University Medical Center, Nijmegen, the Netherlands; 17 Department of Microbiology Tumor and Cell Biology, Karolinska Institutet, Stockholm, Sweden; 18 Division of Parasitic Diseases and Malaria, Center for Global Health, Centers for Disease Control and Prevention, Atlanta, Georgia, USA; 19 Centers for Disease Control and Prevention, Kisumu, Kenya; 20 Grupo Salud y Comunidad, Facultad de Medicina, Universidad de Antioquia, Medellin, Colombia; 21 Department of Microbiology and Immunology, Rijnstate Hospital, Arnhem, the Netherlands; 22 Malaria Research and Training Centre, Faculty of Pharmacy and Faculty of Medicine and Dentistry, University of Science, Techniques, and Technologies of Bamako, Bamako, Mali; 23 Tropical Medicine Research Institute, National Centre for Research, Khartoum, Sudan; 24 Department of Global Health and Infection, Brighton and Sussex Medical School, Brighton, United Kingdom; 25 Kenya Medical Research Institute, Kisian, Kenya; 26 Department of Women’s and Children’s Health, International Maternal and Child Health, Uppsala University, Uppsala, Sweden; 27 Bagamoyo Research and Training Centre, Ifakara Health Institute, Bagamoyo, Tanzania; 28 Africa Academy for Public Health, Dar es Salaam, Tanzania; 29 Department of Parasitology and Medical Entomology, Muhimbili University of Health and Allied Sciences, Dar es Salaam, Tanzania; 30 Department of International Public Health, Liverpool School of Tropical Medicine, Liverpool, United Kingdom; 31 Human Health Division, International Centre for Insect Physiology and Ecology, Mbita Point, Kenya; 32 Department of Biomedical Sciences, Centre National de Recherche et de Formation sur le Paludisme, Ouagadougou, Burkina Faso

**Keywords:** single low-dose primaquine, *Plasmodium falciparum*, gametocytemia

## Abstract

**Background:**

Since the World Health Organization recommended single low-dose (0.25 mg/kg) primaquine (PQ) in combination with artemisinin-based combination therapies (ACTs) in areas of low transmission or artemisinin-resistant Plasmodium *falciparum,* several single-site studies have been conducted to assess efficacy.

**Methods:**

An individual patient meta-analysis to assess gametocytocidal and transmission-blocking efficacy of PQ in combination with different ACTs was conducted. Random effects logistic regression was used to quantify PQ effect on (1) gametocyte carriage in the first 2 weeks post treatment; and (2) the probability of infecting at least 1 mosquito or of a mosquito becoming infected.

**Results:**

In 2574 participants from 14 studies, PQ reduced PCR-determined gametocyte carriage on days 7 and 14, most apparently in patients presenting with gametocytemia on day 0 (odds ratio [OR], 0.22; 95% confidence interval [CI], .17–.28 and OR, 0.12; 95% CI, .08–.16, respectively). Rate of decline in gametocyte carriage was faster when PQ was combined with artemether-lumefantrine (AL) compared to dihydroartemisinin-piperaquine (DP) (*P* = .010 for day 7). Addition of 0.25 mg/kg PQ was associated with near complete prevention of transmission to mosquitoes.

**Conclusions:**

Transmission blocking is achieved with 0.25 mg/kg PQ. Gametocyte persistence and infectivity are lower when PQ is combined with AL compared to DP.

## Teaser

An individual patient meta-analysis was performed on the gametocytocidal and transmission-blocking activities of single-dose primaquine. Gametocyte persistence and infectivity depended on the artemisinin-combination therapy that primaquine was administered with. Primaquine’s transmission-blocking effects were achieved at 0.25 mg/kg.

Antimalarial regimens based on artemisinin and its derivatives, artemisinin-based combination therapies (ACTs) have been adopted widely as first-line treatment of uncomplicated malaria. Despite highly efficient clearance of asexual stage parasites and early gametocytes [1, 2], ACTs do not affect mature *Plasmodium falciparum* gametocytes. Mature gametocytes are responsible for transmission of infection from humans to mosquitoes, and they remain largely unaffected by antimalarial treatment, including ACTs [3–5]. As a result, gametocyte carriage can persist for several days and even weeks after ACT administration [3, 6] and treated individuals can continue to be a source of mosquito infections [3, 7, 8]. As malaria control programs focus their efforts on regional elimination and global eradication and the necessity to contain drug-resistant parasites, targeting gametocytes as part of routine clinical care and community treatment campaigns is being recommended [9–11].

Primaquine (PQ), a drug that is used routinely for the radical cure of *Plasmodium vivax* and *Plasmodium ovale* infections, has been recast as a viable treatment strategy to reduce *P. falciparum* transmission. The ability of PQ and its predecessor plasmoquine to stop *P. falciparum* infectivity to malaria vectors has been known for many decades [12, 13]. In 2012, the World Health Organization (WHO) recommended the use of PQ, in combination with ACTs, in areas approaching elimination and where artemisinin resistance was observed [10]. To mitigate concerns related to hemolysis in individuals with glucose-6-phosphate dehydrogenase (G6PD) deficiency and based on efficacy shown at low doses, a single low dose of 0.25 mg/kg of PQ was recommended for the gametocytocidal indication [10]. The safety of single low-dose PQ was confirmed in subsequent safety studies in individuals with G6PD deficiency [14, 15]. Multiple efficacy studies have been conducted to determine the gametocytocidal and transmission-blocking activity of PQ at different doses and with different partner ACTs.

We conducted a systematic review and individual patient data (IPD) meta-analysis of clinical trials to quantify the ability of single-dose PQ given in combination with different ACTs to clear gametocytes and block transmission, and to compare efficacies of different combinations.

## METHODS

### Data Pooling

Details of the systematic review (PROSPERO CRD42019126710) are provided in the Supplementary Material. Studies were eligible for the inclusion in this analysis if (1) IPD came from a clinical efficacy trial of patients with uncomplicated *P. falciparum* infection or asymptomatic parasite carriers containing at least 1 study arm with a combination of a blood schizonticide and a single dose of PQ; (2) patient demographics and information on dosing (mg/kg) of the blood schizonticide and PQ were available; (3) transmission potential was assessed by weekly gametocyte carriage (ie, prevalence) using molecular methods and/or by membrane feeding assay conducted on day 0 and any day post treatment; and (4) patients were followed up at least until day 14. In the eligible studies, non-ACT study arms, which were randomized to receive PQ or not, were also included in the analysis as they contributed to the overall estimate of PQ effect.

### Ethics

All data included in this analysis were obtained in accordance with the laws and ethical approvals applicable to the countries in which the studies were conducted, and were obtained with the knowledge and consent of the individual to which they relate. Data were fully anonymized either before or during the process of uploading to the WorldWide Antimalarial Resistance Network repository. Use of existing data that are fully anonymized and that researchers cannot trace back to identifiable individuals does not require the review of the Ethics Committee under the guidelines of the Oxford University Research Ethics Committee.

### Statistical Analysis

Statistical analyses were carried out according to an a priori statistical analysis plan [16]. The prevalence of gametocytemia on days 7 and 14 after first administration of any treatment (day 0) was determined separately for patients without and with gametocytes on enrollment. Logistic regression models for gametocyte prevalence (0/1), as measured by molecular methods (quantitative reverse-transcriptase-polymerase chain reaction [qRT-PCR] or quantitative nucleic acid sequence-based amplification [QT-NASBA]), on each day were fitted with random intercepts for study site.

Data from membrane feeding experiments were analyzed using logistic regression to identify predictors of (1) probability of a participant infecting at least 1 mosquito, and (2) probability of a feeding mosquito being infected. Random intercepts were included to account for multiple measurements per patient (1) or clustering within a membrane feeding experiment (2).

Additional details such as predictors considered in each of the regression models and assessment of risk of bias analysis are given in Supplementary Material.

## RESULTS

The systematic review identified 13 studies eligible for inclusion and 2 additional studies were identified in response to the call for data (Supplementary Figure 1). IPD from 14 studies were shared; their details are presented in Supplementary Table 1. Five studies used QT-NASBA (including 2 where quantification was not performed), 8 used qRT-PCR, and 1 study used both. The target transcripts in these molecular assays included *Pfs25*, *Pfs230p,* and *Pfg377* mRNA. In addition to sexual-stage specific parasite detection, 3 of these studies also included data from membrane feeding experiments, where infectiousness was directly quantified by feeding mosquitoes on infected blood and assessing oocyst development 1 week later. G6PD deficiency was assessed using fluorescence spot test in 4 studies, rapid diagnostic test in 5 studies, or genotyping in 3 studies. All studies, except 1 from Colombia, were conducted in Africa at sites with varying transmission intensities. Administration of PQ was randomized and compared to a no-PQ arm in all studies except for 1 in which the dose of PQ was increased sequentially (study 8). A total of 66.7% (1718/2574) of participants received a dose of PQ (25.0%–100.0% in individual studies), of whom 355 (20.7%) were treated on day 0, 1241 (72.2%) on day 2, and 122 (7.1%) on day 3. Of the 1718 individuals treated with PQ, 477 (27.8%) patients received the WHO-recommended 0.25-mg/kg dose and 474 (27.6%) received a 0.40-mg/kg dose. Other doses tested included 0.0625, 0.1, 0.125, 0.2, 0.50, and 0.75 mg/kg (Table 1).

**Table 1.** Baseline Characteristics of Analysis Population^a^

Baseline Characteristics	Primaquine		No Primaquine		All	
	N	Median (Range) or n (%)	N	Median (Range) or n (%)	N	Median (Range) or n (%)
Age, y	1711	9 (0.5–84)	852	9 (1–84)	2563	9 (0.5–84)
Age group						
<5 y	1711	342 (20)	852	162 (19)	2563	504 (20)
5–11 y	1711	799 (47)	852	376 (44)	2563	1175 (46)
12+ y	1711	570 (33)	852	314 (37)	2563	884 (34)
Sex, male	1598	901 (56)	835	472 (57)	2433	1373 (56)
WAZ	328	−0.7 (−3.5 to 2.6)	156	−0.6 (−3.8 to 2.5)	484	−0.7 (−3.8 to 2.6)
Underweight, WAZ <−2	328	38 (12)	156	21 (13)	484	59 (12)
Temperature, °C	1188	36.5 (34.2–40.3)	653	36.7 (34.3–40.4)	1841	36.6 (34.2–40.4)
Fever, >37.5°C	1207	120 (10)	653	119 (18)	1860	239 (13)
Hemoglobin, g/dL	1688	11.7 (6–18.7)	837	11.7 (6.8–17.8)	2525	11.7 (6–18.7)
Anemia, Hb < 10 g/dL	1688	240 (14)	837	126 (15)	2525	366 (14)
Parasitemia, /µL	1618	560 (0–518 180)	774	1000 (0–432 000)	2392	687.5 (0–518 180)
Hyperparasitemia, >10^5^/µL	1618	103 (6)	774	36 (5)	2392	139 (6)
Presence of gametocytes						
Microscope	833	212 (25)	491	162 (33)	1324	375 (28)
QT-NASBA	1215	925 (76)	501	385 (77)	1716	1310 (76)
RT-PCR	525	408 (76)	410	407 (75)	945	715 (76)
Gametocytemia, /µL						
Microscope	132	64 (12–1136)	133	43 (16–3000)	265	48 (12–3000)
QT-NASBA	871	22.7 (0–32 733.6)	376	32.1 (0–17 944.5)	1247	25.7 (0–32 733.6)
RT-PCR	249	29.6 (0–4988.8)	172	31.7 (0–6529.5)	421	30.5 (0–6529.5)
G6PD deficient	1581	96 (6)	743	49 (7)	2324	145 (6)
Treatment administered						
Schizontal treatment						
AL	1718	858 (50)	856	420 (49)	2574	1278 (50)
ASSP	1718	106 (6)	856	106 (12)	2574	212 (8)
DP	1718	734 (43)	856	310 (36)	2574	1044 (41)
SPAQ	1718	20 (1)	856	20 (2)	2574	40 (2)
Dose of primaquine, mg/kg						
0.0625	1718	16 (1)	…	…	…	…
0.100	1718	115 (7)	…	…	…	…
0.125	1718	25 (1)	…	…	…	…
0.200	1718	172 (10)	…	…	…	…
0.250	1718	477 (28)	…	…	…	…
0.400	1718	474 (28)	…	…	…	…
0.500	1718	17 (1)	…	…	…	…
0.750	1718	422 (25)	…	…	…	…

Abbreviations: AL, artemether-lumefantrine; ASSP, artesunate and sulfadoxine-pyrimethamine; DP, dihydroartemisinin-piperaquine; G6PD, glucose-6-phosphate dehydrogenase; Hb, hemoglobin; N, number of patients evaluated; n, number of patients in that category; QT-NASBA, quantitative nucleic acid sequence-based amplification; RT-PCR, reverse transcription polymerase chain reaction; SPAQ, sulfadoxine-pyrimethamine and amodiaquine; WAZ, weight-for-age score.

^a^Includes 20 patients who received DP and methylene blue and only contributed baseline data from membrane feeding experiments.

The median age of study participants was 9 years (interquartile range [IQR], 5–14) with 19.7% (504/2563) younger than 5 years. Most of the 2574 study participants were treated with artemether-lumefantrine (AL) (1278; 49.7%) or dihydroartemisinin-piperaquine (DP) (1044; 40.7%). Other treatments administered included: artesunate-sulfadoxine-pyrimethamine (ASSP) (212; 8.3%) and sulfadoxine-pyrimethamine-amodiaquine (SPAQ) (40; 1.6%). At enrolment, 14.5% (366/2525) of patients presented with anemia (hemoglobin level below 10.0 g/dL), 12.8% (239/1860) with fever, and 5.8% (139/2392) had more than 100 000 parasites/µL (Table 1); 12.2% (59/484) of the children <5 years of age were underweight (weight-for-age z-score < −2). The proportion of participants with fever at enrolment was lower in the group of individuals receiving PQ compared to the group that did not receive PQ (9.9% vs 18.2%, respectively); however, the difference was not significant after adjusting for study site (*P* = .966). Six studies’ protocols excluded individuals with G6PD deficiency (Supplementary Table 1).

### Gametocytemia After Treatment in Participants With No Detectable Gametocytes at Baseline

In total, 632 (31.3%) patients presented without detectable gametocytes on enrolment, of whom 481 (76.1%) were assessed weekly for gametocyte carriage during the first 14 days of follow-up. Detectable posttreatment gametocytemia was present in 12.9% (39/302) of patients treated with PQ compared to 19.6% (35/179) of those not treated with PQ (odds ratio [OR], 0.55; confidence interval [95% CI], .32–.96; *P* = .035, adjusted for study-site random effect) (Supplementary Table 2). The effect of PQ on gametocyte appearance was similar (*P* = .308) between day 7 (OR, 0.58; 95% CI, .33–1.01; *P* = .053) and day 14 (OR, 0.30; 95% CI, .14–.63; *P* = .002).

### Gametocytemia After Treatment in Participants With Gametocytes at Baseline

**Figure 1.**
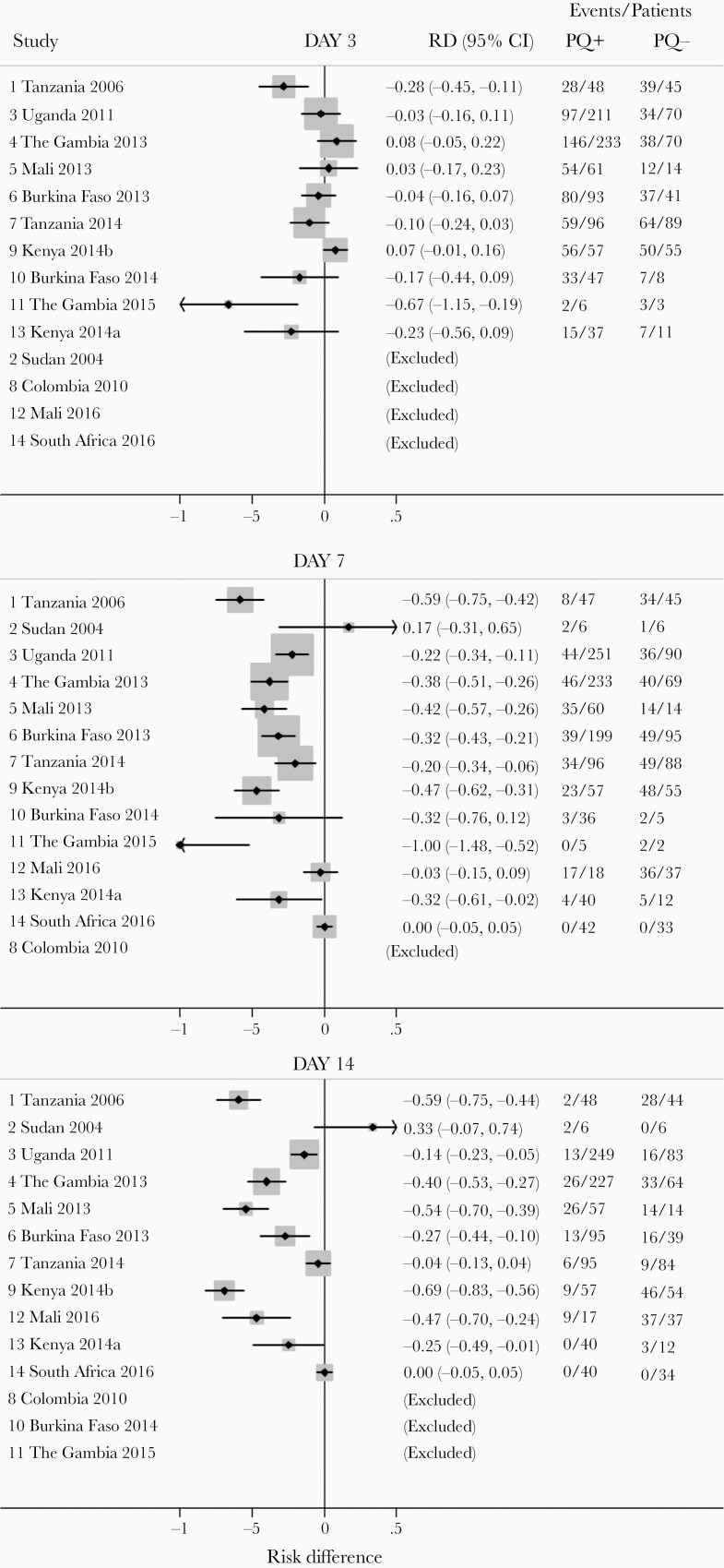
Forest plots of difference in proportions of participants with gametocytes (risk difference) on each day of follow-up. Only individuals with gametocytes at enrolment were included. Day 3, heterogeneity χ ^2^ = 14.90 (df = 8); *P* = .061; *I*^2^ = 46.3. Day 7, heterogeneity χ ^2^ = 45.75 (df = 8); *P* < .001; *I*^2^ = 82.5%. Day 14, heterogeneity χ ^2^ = 70.21 (df = 8); *P* < .001; *I*^2^ = 88.6%. Studies were excluded if no data were collected on a specific day, except for study 8, which only included PQ arms (all days), and study 14 (day 3) in which PQ was administered on day 3. Abbreviations: CI, confidence interval; PQ, primaquine; RD, risk difference.

At enrolment, 1754 (68.7%) patients had gametocytes detected by molecular methods. Among those patients treated with PQ, 23.4% (258/1101) had gametocytes detected on day 7 compared to 57.4% (316/551) of those not treated with PQ (OR, 0.22; 95% CI, .17–.28; *P* < .001). The corresponding proportions of individuals who were still gametocytemic on day 14 were 11.4% (106/931) and 42.9% (202/471), respectively (OR, 0.12; 95% CI, .08–.16; *P* < .001) (Supplementary Table 2 and Figure 1). In multivariable mixed effects models, gametocyte positivity on day 7 was associated significantly with gametocyte and asexual parasite densities and hemoglobin concentration at baseline (Table 2). Compared to patients treated with DP, those treated with AL were significantly less likely to have gametocytes on day 7 (adjusted OR [AOR], 0.50; 95% CI, .28–.90; *P* = .021), while those treated with SPAQ were more likely to carry gametocytes (AOR, 16.16; 95% CI, 1.88–139; *P* = .011). On day 14, only the baseline gametocyte density and antimalarial treatment were associated with gametocyte carriage. After adjustment for these factors, a higher dose of PQ was associated with lower prevalence of gametocyte positivity on days 7 and 14 (AOR, 0.69; 95% CI, .65–.74 and AOR, 0.58; 95% CI, .53–.64 for each 0.1-mg/kg increase in dose, respectively; both *P* < .001). This dose effect translates to an AOR of 0.40 (95% CI, .34–.46) for day 7 gametocyte carriage and AOR 0.26 (95% CI, .20–.33) for day 14 gametocyte carriage for patients who received 0.25-mg/kg dose of PQ compared to those who did not receive PQ.

**Table 2.** Multivariable Mixed Effects Logistic Regression for Gametocyte Positivity^a^ on Days 7 and 14 in Patients With Detectable Gametocytemia on Day 0.

Parameter	Day 7 Gametocyte Positivity (N = 1509, n = 546)			Day 14 Gametocyte Positivity (N = 1316, n = 306)		
	AOR	95% CI	*P* Value	AOR	95% CI	*P* Value
PQ dose per 0.1 mg/kg	0.69	.65–.74	<.001	0.58	.53–.64	<.001
Log_10_ gametocytemia^b^	1.85	1.61–2.13	<.001	1.87	1.56–2.25	<.001
Hyperparasitemia, >10^5^ parasites/µL	0.28	.15–.53	<.001			
Hemoglobin, g/dL	0.85	.78–.92	<.001			
Treatment						
DP	Reference			Reference		
AL	0.50	.28–.90	.021	0.18	.08–.44	<.001
ASSP	1.20	.45–3.21	.723	0.99	.26–3.80	.983
SPAQ	16.16	1.88–138.70	.011	1.30	.30–5.72	.726

Abbreviations: AL, artemether-lumefantrine; AOR, adjusted odds ratio; ASSP, artesunate and suphadoxine-pyrimethamine; CI, confidence interval; DP, dihydroartemisinin-piperaquine; N, number of patients included in the model; n, number of patients with positive outcome; PQ, primaquine; qRT-PCR, quantitative reverse transcription polymerase chain reaction; SPAQ, sulfadoxine-pyrimethamine and amodiaquine.

^a^When results from both molecular methods were available, gametocyte density was defined by qRT-PCR.

^b^In studies where only gametocyte positivity was determined by a molecular method, density measures by microscopy were included. For patients with positive samples by molecular method and zero microscopy count (n = 230 on day 7 and n = 180 on day 14), density was assumed to be 8 (half of the detection limit by microscopy assuming microscopic quantification against 500 white blood cells or 1/16th of a microliter).

**Figure 2.**
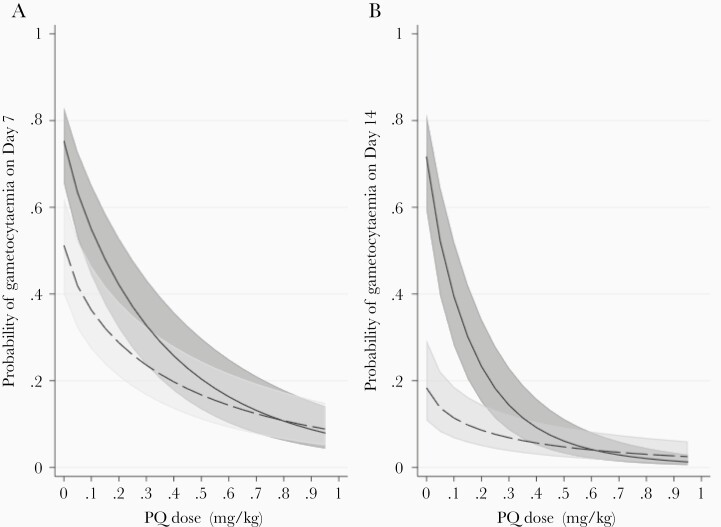
Predicted relationship between probability of gametocyte carriage on days 7 (*A*) and 14 (*B*) post treatment initiation and PQ dose. The dashed line represents this relationship for individuals treated with AL and the solid line for individuals treated with DP. Shaded areas correspond to 95% confidence intervals. Median values for other variables were assumed. Abbreviations: AL, artemether-lumefantrine; DP, dihydroartemisinin-piperaquine; PQ, primaquine.

A fractional polynomial model was used to estimate the probability of gametocyte carriage on days 7 and 14 for 1543 individuals receiving different PQ doses with AL or DP (Figure 2). Whilst addition of PQ reduced gametocyte carriage for both ACTs, the rate of decline in gametocyte carriage associated with PQ dose differed between patients treated with AL and DP (test for interaction, *P* = .010 for day 7 and *P* < .001 for day 14). Among individuals treated with AL, most of the reduction in gametocyte carriage probability was achieved with the recommended 0.25-mg/kg PQ dose, whereas in individuals treated with DP higher doses of PQ were associated with additional substantial reductions in gametocyte carriage. Administration of a PQ dose of 0.25 mg/kg in patients treated with AL reduced risk of gametocytemia on day 7 to 26.0% (95% CI, 18.7%–34.9%) and on day 14 to 7.6% (95% CI, 4.3%–13.2%) compared to 37.1% (95% CI, 27.6%–47.8%) and 18.2% (95% CI, 11.4%–27.9%) in patients treated with DP, respectively.

The risk for gametocyte carriage was significantly higher on day 7 in patients treated with PQ on day 2 or 3 compared to patients treated with PQ on day 0 (AOR, 2.28; 95% CI, 1.66–3.69; *P* < .001, adjusted for covariates in the main analysis; Table 2). However, this difference was not statistically significant by day 14 (AOR, 1.74; 95% CI, .80–3.81; *P* = .164, adjusted for covariates in the main analysis; Table 2).

Administration of PQ also reduced gametocyte density in those positive on days 7 or 14. Expressed as a proportion of the baseline gametocyte density, gametocyte densities reached median values of 2.0% (interquartile range [IQR], 0.3%–10.2%) relative to baseline by day 7 in PQ-treated individuals compared to 29.8% (IQR, 8.1%–77.4%) in individuals who did not receive PQ (*P* < .001 Wald test, adjusted for ACT and study). The corresponding values on day 14 were 0.5% (IQR, 0.1%–5.6%) in PQ-treated individuals and 9.6% (IQR, 1.5%–36.0%) in individuals who did not receive PQ (*P* < .001, Wald test adjusted for ACT and study).

### Mosquito Feeding Assays

In the 3 studies undertaking mosquito feeding experiments (Supplementary Table 1 and Supplementary Table 3), participants were treated with either AL (1 study), DP (2 studies), or SPAQ (1 study) and a PQ dose of 0.25 mg/kg was compared to ACT alone in all studies. In 1 of these studies, the 0.40-mg/kg dose was tested, and in another study, PQ doses of 0.0625, 0.125, and 0.50 mg/kg were also administered. These data are presented in Supplementary Table 4.

**Figure 3.**
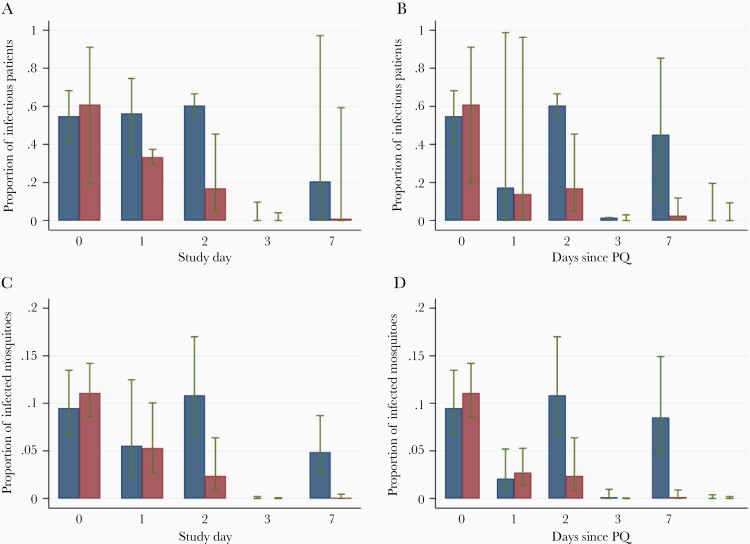
Results of membrane feeding experiments on different days of follow-up, in relation to starting treatment (*A* and *C*) and time of PQ administration (*B* and *D*). Whiskers represent 95% confidence intervals adjusted for clustering within patients (*A* and *B*) and within feeding experiments (*C* and *D*). Red boxes represent data for PQ arms and blue boxes for arms without PQ administration. This figure includes all data combined from AL, DP, and SPAQ treatment arms. Abbreviations: AL, artemether-lumefantrine; DP, dihydroartemisinin-piperaquine; PQ, primaquine; SPAQ, sulfadoxine-pyrimethamine and amodiaquine.

Among 316 feeding experiments conducted prior to treatment on participants with baseline gametocytemia, 186 (58.9%) infected at least 1 mosquito, with a median of 13.9% (range, 1.2%–96.5%) of mosquitoes infected (Figure 3 and Supplementary Table 4). While the proportion of the infected mosquitoes (in infectious feeds) was similar between the 3 studies (*P* = .369), the number of noninfectious feeds ranged from 37.8% to 67.9% (*P* < .001) between studies, with the lowest proportion observed in study 6 (AL/AL + PQ). This study had the lowest baseline gametocytes levels; 79.0% of patients had fewer than 50 gametocytes/µL compared to 24.7% and 42.5% in the other 2 studies.

In patients with confirmed gametocytemia at baseline and at the time of sampling post treatment, 13.2% of feeds (36/272) of those treated with PQ infected at least 1 mosquito, compared to 35.6% (63/177) of non-PQ–treated patients sampled at the same timepoints (Figure 3 and Supplementary Table 4). There were significant differences between studies/treatments: among patients who did not receive PQ, only 1 feed (1/61, 1.6%; days tested 3, 7, 10, and 14) infected any mosquitoes after AL compared to 49.4% (39/79; days tested 1, 2, and 7) for DP and 59.0% (23/39; days tested 1, 2, 6, 7, and 8) for SPAQ. In the PQ arms, the proportion of feeds that infected any mosquitoes was 0.0% (0/83) with AL, 2.6% (1/38) with SPAQ, and 22.2% (35/158) with DP. From day 5 after PQ administration, of 283 feeds only 2 feeds were infectious, both in DP arms with PQ doses of 0.0625 and 0.5 mg/kg.

**Figure 4.**
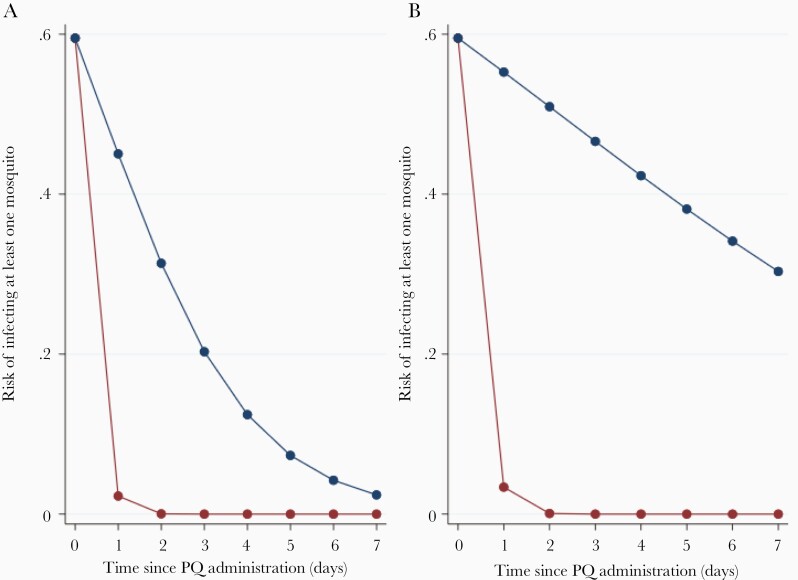
Predicted risk of infecting at least 1 mosquito in the membrane feeding experiment, after administration of 0.25-mg/kg dose of PQ (red line) or without PQ administration (blue line). Gametocytemia of 100 gametocytes per microliter was assumed at the time of sampling. Results are presented for patients treated with AL (*A*) or DP (*B*). Abbreviations: AL, artemether-lumefantrine; DP, dihydroartemisinin-piperaquine; PQ, primaquine.

The risk of a participant infecting at least 1 mosquito and the risk of a feeding mosquito becoming infected were strongly associated with gametocyte density at the time of mosquito feeding (AOR, 8.33; 95% CI, 3.91–17.78 and AOR, 6.58; 95% CI, 4.16–10.40 for 10-fold increases in gametocyte density, respectively) and significantly decreased following PQ treatment (Table 3). The reduction in odds of mosquito infectivity over time associated with PQ dose of 0.25 mg/kg was significantly higher compared to lower doses (0.0625–0.125 mg/kg) (ratio of AORs per day, 17.84; 95% CI, 4.93–64.52; *P* < .001 for a participant infecting at least 1 mosquito and 10.36; 95% CI, 4.67–22.98; *P* < .001 for a mosquito becoming infected) and not statistically different from higher doses (0.4–0.5 mg/kg) (*P* = .433 and *P* = .070, respectively). With the exception of those treated with AL, the odds did not decrease significantly over time for any of the schizontocidal drugs. A PQ dose of 0.25 mg/kg decreased the risk of infecting at least 1 mosquito practically to zero by day 3 (Figure 4 and Supplementary Figure 2).

**Table 3.** Multivariable Mixed Effects Logistic Regression for Probability of a Patient Infecting at Least 1 Mosquito and Probability of a Mosquito Being Infected in Membrane Experiments Conducted on Blood Taken Within 14 Days From Treatment in Patients With Gametocytemia at Baseline and at the Time of Sampling

Parameter	Patient Infecting at Least 1 Mosquito (N = 317 Patients, n = 684 Feeds)			Mosquito Gets Infected (N = 41 840 Mosquitoes, n = 664 Feeds, 317 Patients)		
	AOR^a^	95% CI	*P* Value	AOR^a^	95% CI	*P* Value
Effect of PQ dose over time, per day						
0.0625–0.125 mg/kg	0.50	.31–.81	.004	0.57	.41–.70	.001
0.25 mg/kg	0.03	.01–.11	<.001	0.05	.03–.12	<.001
0.4–0.5 mg/kg	0.06	.01–.32	.001	0.18	.06–.54	.002
Effect of treatment over time, per day						
AL	0.56	.36–.87	.010	0.52	.37–.73	<.001
DP	0.84	.69–1.02	.082	0.96	.83–1.11	.593
SPAQ	0.97	.76–1.23	.798	0.98	.83–1.16	.807
Log_10_ gametocytemia at the time of sampling	8.33	3.91–17.78	<.001	6.58	4.16–10.40	<.001

Abbreviations: AL, artemether-lumefantrine; AOR, adjusted odds ratio; CI, confidence interval; DP, dihydroartemisinin-piperaquine; PQ, primaquine; SPAQ, sulfadoxine-pyrimethamine and amodiaquine.

^a^Estimates also adjusted for study included as a covariate

### Risk of Bias

Methodological factors potentially contributing to the risk bias and attrition bias are presented in Supplementary Table 5. Measurement of gametocyte carriage using molecular methods is automated, minimizing the risk of observer bias; laboratory personnel performing molecular assays or dissecting mosquitoes were blinded in all studies. Sensitivity analyses showed that exclusion of any of the studies did not change the main conclusions of the analysis. The effect of PQ dose on gametocyte positivity was estimated as median AOR 0.69 (range, 0.65–0.70) on day 7 and 0.58 (range, 0.54–0.62) on day 14 for a 0.1-mg/kg increase.

The only eligible study for which data were not available for this meta-analysis [8] presented similar findings to results of this analysis. In this study, the addition of a single dose of 45 mg of PQ to DP treatment was associated with increased clearance of gametocytes (measured by PCR) on day 7 and day 14. In the PQ arm, of 24 patients with gametocytes on enrolment, 22 cleared gametocytemia by day 7 and all by day 14, compared to 11 (day 7) and 16 (day 14) of the 22 patients in the DP only arm. In their membrane feeding experiments, no mosquito infections occurred in the PQ arm 1 and 2 weeks post treatment, while in the no-PQ arm 6.9% of feeding mosquitoes were infected on day 7 and 5.0% on day 14.

## DISCUSSION

This IPD meta-analysis estimated the effect of PQ as a single dose (ranging from 0.0625 to 0.75 mg/kg) on the transmission potential of falciparum malaria infections, when coadministered with schizonticidal drugs. Our findings confirm the gametocyte clearing and sterilizing effects of single-dose PQ and indicate that both the PQ and the schizonticidal partner drug are important determinants of gametocyte clearance and transmission potential. Regardless of the schizonticidal partner drug, mosquito infections were rarely observed 1 week after administration of PQ; however, only 3 of the 14 studies contributed data to this analysis.

Among currently licensed antimalarials for *P. falciparum*, PQ is unique in its ability to clear mature gametocytes persisting after ACT treatment. Because the impact of ACTs is largely restricted to immature, developing gametocytes [17], only a small proportion of infections develop gametocytes after ACTs whilst gametocytes that are present prior to treatment may persist [6]. In the current analysis, more than 20% of individuals who were gametocyte negative at enrolment became gametocyte positive by molecular gametocyte detection methods shortly after treatment. Given that gametocytes first appear 8.5–12 days after their asexual progenitors [18] and transcripts specific to mature gametocytes are first observed on day 3 based on the current data, it is likely that this reflects density fluctuations of mature gametocytes already present prior to treatment [19], rather than de novo gametocyte production. In line with this, PQ administration prior to first detection of gametocytes reduced the proportion of patients with gametocytes during follow-up.

Gametocyte kinetics in patients who presented with peripheral gametocytemia were strongly dependent on the schizontocidal treatment administered. Non-ACTs leave gametocytes largely unaffected, with gametocyte kinetics resembling a natural decay, while ACTs are only effective against early gametocytes [2, 20]. Also, ACTs differ markedly in their impact on gametocyte carriage [6, 7, 21], potentially due to the effects of the nonartemisinin partner drugs. Whilst lumefantrine affects gametocytes and their infectivity [22], piperaquine has limited effect on either developing or mature gametocytes [23]. Furthermore, the artemisinin derivative dose recommended by the manufacturer is significantly higher for AL than for DP. In the current pooled analysis, individuals receiving AL were considerably less likely to have gametocytemia on day 14 compared to DP (AOR, 0.18; 95% CI, .08–.44) and considerably less likely to infect mosquitoes. The addition of PQ significantly reduced gametocyte carriage in all treatment groups [24] and did so in a dose-dependent manner [25]. When given in combination with AL, the 0.25-mg/kg WHO-recommended dose reduced gametocyte prevalence 7 days after treatment initiation to 22%, and this reduction is similar to that observed for higher PQ doses (16%, *P* = .202). For individuals receiving DP, the average gametocyte prevalence reduction for 0.25 mg/kg PQ was only to 39% on day 7 post treatment but higher PQ doses accelerated gametocyte clearance (to 15%, *P* = .002), and a 0.40-mg/kg PQ dose coadministered with DP achieves a similar effect to a 0.25-mg/kg dose coadministered with AL.

However, gametocyte sterilization may precede gametocyte clearance [26, 27]. In 3 studies included where mosquito infection was used as an endpoint, the effect of PQ on preventing mosquito infection was apparent before gametocytes were fully cleared. Whilst the gametocyte clearing effect of PQ only became apparent on day 7 post initiation of treatment, mosquito infections were already very rare on day 3 following treatment with 0.25 mg/kg PQ. PQ doses below 0.25 mg/kg were associated with higher mosquito infection rates on day 3 whilst doses higher than 0.25 mg/kg did not augment or accelerate the transmission-blocking properties of PQ.

Use scenarios for single-dose PQ include elimination settings and areas threatened by drug resistance [10]. The findings from this meta-analysis, of increased gametocyte clearance and near absence of mosquito infections after administration (only 10/220 individuals who received at least 0.25 mg/kg PQ infected mosquitoes in feeding assays), support PQ deployment in these scenarios. PQ has been coadministered with schizonticides in community-wide treatment campaigns [9, 28, 29], on the assumption that asymptomatic infections constitute a substantial proportion of the human infectious reservoir for malaria in low-endemic settings [30, 31]. However, concerns have been raised regarding the risk to benefit ratio in these settings. A proportion of these populations are likely to be G6PD deficient with a concern that they may be at an increased risk of PQ-induced hemolysis. The WHO-recommended single low dose of PQ has shown no significant risk in recent studies specifically designed to assess safety in this population [14, 15], nor in recent studies primarily designed to determine PQ efficacy [32–34]. Results of an IPD meta-analysis of all available safety data will be published separately (PROSPERO CRD42019128185).

While CYP2D6 activity is essential for the generation of metabolites implicated in hypnozoite-clearance in *P. vivax* [35, 36], less is known about its potential impact on gametocytocidal or transmission-blocking properties of PQ. Whilst PQ’s gametocytocidal activity may in part be unrelated to cytochrome CYP2D6 activity [36], gametocytes may persist longer after PQ treatment in individuals with low-moderate CYP2D6 activity [37]. A shortcoming of our meta-analysis is that we could not incorporate these possible effects of CYP2D6 metabolizer status on post-PQ gametocyte carriage or transmission. In general, the added value of gametocytocidal drugs in community treatment campaigns continues to be a matter of debate. Mathematical simulations indicate that the fraction of the asymptomatic population that is successfully treated with ACTs is considerably more important for the impact of treatment campaigns than the addition of PQ to ACTs and that impact will depend on transmission intensity [38–40].

This study also highlights SPAQ’s poor ability to clear gametocytes with a considerably higher gametocyte prevalence on day 7 post initiation of treatment compared to DP or AL [41]. Seasonal malaria chemoprevention (SMC) using SPAQ is widely deployed across the Sahel region of Africa to reduce malaria morbidity in children younger than 5 years and consists of giving all children SPAQ 3 to 4 times monthly during the transmission season. In scenarios where SMC campaigns are considered in wider age groups, SMC may impact gametocyte carriage [42] and malaria transmission. For such scenarios, our findings suggest that either adding single low-dose PQ to SPAQ or changing to an artemisinin-based combination of drugs may increase SMC impact [3].

## CONCLUSIONS

Our analysis, based on IPD from clinical trials that were primarily conducted in Africa, supports the use of PQ as a potent gametocytocide and transmission-blocking tool for *P. falciparum* malaria. Gametocyte carriage and transmission after PQ treatment depend on the schizonticidal drug that PQ is combined with, and PQ doses higher than 0.25 mg/kg may accelerate gametocyte clearance. However, this WHO-recommended dose effectively achieves near-complete reductions in mosquito infections regardless of ACT. Additional clinical trials are necessary to quantify the effect of PQ use at community level; that is, to determine whether the effect of PQ observed in mosquito feeding assays leads to detectable changes in community-wide transmission levels when the drug is systematically used in clusters of transmission.

## Supplementary Data

Supplementary materials are available at *The Journal of Infectious Diseases* online. Consisting of data provided by the authors to benefit the reader, the posted materials are not copyedited and are the sole responsibility of the authors, so questions or comments should be addressed to the corresponding author.

jiaa498_suppl_Supplementary_Table_1

jiaa498_suppl_Supplementary_Table_2

jiaa498_suppl_Supplementary_Table_3

jiaa498_suppl_Supplementary_Table_4

jiaa498_suppl_Supplementary_Table_5

jiaa498_suppl_Supplementary_Figure_1

jiaa498_suppl_Supplementary_Figure_2

jiaa498_suppl_Supplementary_Methods

## Notes

***Acknowledgments.*** The authors thank all participating patients and everyone involved in the original studies. We also thank Caitlin Richmond for project management support.

***Author contributions.*** A. D., C. J. D., I. C., P. J. G., R. G., and T. B. conceived the study. A. M. S., A. D., A. C. E., A. E. M., A. B., A. M., B. B. E. S., B. E. N., B. P. G., C. C. D., D. M., G. J. B., I. I. C., J. R., J. M. B., J. O., K. I. B., M. I. A. A., M. R. S., M. E. R., P. S., R. R. M., R. G., S. E. G. E., S. K., T. B., T. K. K., U. D. A., and W. J. R. S. conceived and undertook individual studies and enrolled patients. G. S. H., I. C., E. C., and K. S. conducted systematic review and data pooling. K. S., T. B., B. P. G., C. J. D., I. C., K. I. B., P. J. G., R. N. P., and R. G. analyzed the data and interpreted the results. B. P. G., K. S., and T. B. wrote the first draft of the manuscript. All authors reviewed and edited the manuscript, and read and approved the final manuscript.

***Financial support.*** This work was supported by the Bill and Melinda Gates Foundation (grant number OPP1089413). The WorldWide Antimalarial Resistance Network is funded by the Bill and Melinda Gates Foundation and the ExxonMobil Foundation.

***Potential conflicts of interest.*** U. D. A. attended Sanofi meeting in Dakar, 13–14 December 2019, and received consultancy fee, travel, and accommodation reimbursement. All other authors report no potential conflicts. All authors have submitted the ICMJE Form for Disclosure of Potential Conflicts of Interest. Conflicts that the editors consider relevant to the content of the manuscript have been disclosed.
