# Supplementary material for: Efficacy of Single-Dose Primaquine With Artemisinin Combination Therapy on Plasmodium falciparum Gametocytes and Transmission: An Individual Patient Meta-Analysis
Source: J Infect Dis. 2020 Aug 11;225(7):1215–26. doi: 10.1093/infdis/jiaa498 (PMC8974839; doi:10.1093/infdis/jiaa498)
Supplement: jiaa498_suppl_Supplementary_Table_2 [file jiaa498_suppl_supplementary_table_2.docx]

Supplementary Table 2. Gametocyte positivity by follow-up day. Any of the molecular methods was used to determine sexual-stage carriage.

| *Participants with gametocytes at enrolment* | | | | | | | |
| --- | --- | --- | --- | --- | --- | --- | --- |
|  |  | **Primaquine** | | | **No Primaquine** | | |
|  |  | **N examined** | **N positive** | **% positive** | **N examined** | **N positive** | **% positive** |
|  |  |  |  |  |  |  |  |
|  | **Day 0** | 1,179 | 1,179 | 100 | 575 | 575 | 100 |
|  | **Day 3**^a^ | 900 | 576 | 64 | 406 | 291 | 72 |
|  | **Day 7** | 1,101 | 258 | 23 | 551 | 316 | 58 |
|  | **Day 14** | 931 | 106 | 11 | 471 | 202 | 43 |
|  |  |  |  |  |  |  |  |
| *Participants with no detectable gametocytes at enrolment* | | | | | | | |
|  |  | **Primaquine** | | | **No Primaquine** | | |
|  |  | **N examined** | **N positive** | **% positive** | **N examined** | **N positive** | **% positive** |
|  | **Day 0** | 415 | 0 | 0 | 217 | 0 | 0 |
|  | **Day 3**^a^ | 287 | 61 | 21 | 127 | 38 | 30 |
|  | **Day 7** | 383 | 36 | 9 | 203 | 31 | 15 |
|  | **Day 14** | 311 | 13 | 4 | 185 | 20 | 11 |
|  |  |  |  |  |  |  |  |

^a^excludes study (Study ID 14) with primaquine administered at 72h
