# Supplementary material for: Efficacy of Single-Dose Primaquine With Artemisinin Combination Therapy on Plasmodium falciparum Gametocytes and Transmission: An Individual Patient Meta-Analysis
Source: J Infect Dis. 2020 Aug 11;225(7):1215–26. doi: 10.1093/infdis/jiaa498 (PMC8974839; doi:10.1093/infdis/jiaa498)
Supplement: jiaa498_suppl_Supplementary_Table_4 [file jiaa498_suppl_supplementary_table_4.docx]

Supplementary Table 4. Results from membrane feeding experiments.

|  |  |  |  |  | Patients | |  | Mosquitoes | |  | Proportion of infected mosquitoes/patient | | |
| --- | --- | --- | --- | --- | --- | --- | --- | --- | --- | --- | --- | --- | --- |
| Study ID | Treatment arm | PQ arm (mg/kg) | Study Day | Days since PQ | N tested | N infectious | % | N tested | N infected | % | Median | IQR | Range |
| 6 | AL | 0 | 0 | 0 | 32 | 15 | 47 | 1402 | 207 | 15 | 28 | [9-48] | [3-92] |
|  |  | 0 | 3 | 0 | 36 | 0 | 0 | 1609 | 0 | 0 |  |  |  |
|  |  | 0 | 7 | 0 | 66 | 1 | 2 | 2885 | 4 | 0 | 9 | [9-9] | [9-9] |
|  |  | 0 | 10 | 0 | 17 | 0 | 0 | 822 | 0 | 0 |  |  |  |
|  |  | 0 | 14 | 0 | 17 | 0 | 0 | 855 | 0 | 0 |  |  |  |
|  |  | 0.25 | 0 | 0 | 27 | 8 | 30 | 1195 | 54 | 5 | 14 | [5-24] | [2-49] |
|  |  | 0.25 | 3 | 1 | 41 | 0 | 0 | 1869 | 0 | 0 |  |  |  |
|  |  | 0.25 | 7 | 5 | 67 | 0 | 0 | 2956 | 0 | 0 |  |  |  |
|  |  | 0.25 | 10 | 8 | 18 | 0 | 0 | 848 | 0 | 0 |  |  |  |
|  |  | 0.25 | 14 | 12 | 18 | 0 | 0 | 890 | 0 | 0 |  |  |  |
|  |  | 0.4 | 0 | 0 | 20 | 7 | 35 | 897 | 80 | 9 | 20 | [7-51] | [7-56] |
|  |  | 0.4 | 3 | 1 | 47 | 0 | 0 | 2143 | 0 | 0 |  |  |  |
|  |  | 0.4 | 7 | 5 | 63 | 0 | 0 | 2783 | 0 | 0 |  |  |  |
|  |  | 0.4 | 10 | 8 | 19 | 0 | 0 | 890 | 0 | 0 |  |  |  |
|  |  | 0.4 | 14 | 12 | 17 | 0 | 0 | 841 | 0 | 0 |  |  |  |
| 5 | DP | 0 | 0 | 0 | 32 | 18 | 56 | 2008 | 127 | 9 | 9 | [3-15] | [1-40] |
|  |  | 0 | 1 | 0 | 15 | 8 | 53 | 890 | 53 | 5 | 7 | [4-18] | [2-28] |
|  |  | 0 | 2 | 0 | 14 | 8 | 57 | 951 | 74 | 6 | 7 | [5-25] | [2-32] |
|  |  | 0 | 7 | 0 | 14 | 3 | 21 | 1021 | 35 | 4 | 20 | [2-24] | [2-24] |
|  |  | 0.065 | 0 | 0 | 32 | 22 | 69 | 2103 | 390 | 19 | 21 | [6-48] | [1-91] |
|  |  | 0.065 | 1 | 1 | 16 | 10 | 63 | 1059 | 73 | 7 | 8 | [3-22] | [1-31] |
|  |  | 0.065 | 2 | 2 | 16 | 8 | 50 | 1138 | 56 | 5 | 4 | [2-15] | [1-34] |
|  |  | 0.065 | 7 | 7 | 15 | 1 | 7 | 1080 | 8 | 1 | 11 | [11-11] | [11-11] |
|  |  | 0.125 | 0 | 0 | 34 | 16 | 47 | 2287 | 236 | 10 | 18 | [5-29 | [1-55] |
|  |  | 0.125 | 1 | 1 | 16 | 4 | 25 | 1031 | 63 | 7 | 17 | [9-38] | [1-60] |
|  |  | 0.125 | 2 | 2 | 16 | 3 | 19 | 1034 | 9 | 1 | 7 | [1-9] | [1-9] |
|  |  | 0.125 | 7 | 7 | 15 | 0 | 0 | 1026 | 0 | 0 |  |  |  |
|  |  | 0.25 | 0 | 0 | 30 | 28 | 93 | 2258 | 801 | 37 | 33 | [11-60] | [1-97] |
|  |  | 0.25 | 1 | 1 | 13 | 5 | 38 | 968 | 62 | 6 | 2 | [1-4] | [1-73] |
|  |  | 0.25 | 2 | 2 | 15 | 1 | 7 | 1112 | 7 | 1 | 10 | [10-10] | [10-10] |
|  |  | 0.25 | 7 | 7 | 15 | 0 | 0 | 1082 | 0 | 0 |  |  |  |
|  |  | 0.5 | 0 | 0 | 34 | 20 | 59 | 2192 | 210 | 9 | 8 | [2-17] | [1-60] |
|  |  | 0.5 | 1 | 1 | 17 | 2 | 12 | 1083 | 26 | 2 | 20 | [1-39] | [1-39] |
|  |  | 0.5 | 2 | 2 | 17 | 1 | 6 | 1154 | 3 | 0 | 4 | [4-4] | [4-4] |
|  |  | 0.5 | 7 | 7 | 17 | 1 | 6 | 1145 | 1 | 0 | 1 | [1-1] | [1-1] |
| 12 | DP | 0 | 0 | 0 | 40 | 23 | 58 | 3117 | 318 | 10 | 10 | [3-23] | [1-68] |
|  |  | 0 | 2 | 0 | 20 | 13 | 65 | 1522 | 142 | 9 | 11 | [3-23] | [1-57] |
|  |  | 0 | 7 | 0 | 18 | 9 | 50 | 1340 | 115 | 9 | 10 | [6-12] | [3-67] |
| 12 | SPAQ | 0 | 0 | 0 | 20 | 12 | 60 | 1517 | 170 | 11 | 8 | [5-19] | [1-94] |
|  |  | 0 | 1 | 0 | 1 | 1 | 100 | 82 | 1 | 1 | 1 | [1-1] | [1-1] |
|  |  | 0 | 2 | 0 | 19 | 11 | 58 | 1484 | 213 | 14 | 17 | [7-33] | [1-96] |
|  |  | 0 | 7 | 0 | 19 | 11 | 58 | 1370 | 168 | 12 | 6 | [1-52] | [1-86] |
|  |  | 0.25 | 0 | 0 | 20 | 19 | 95 | 1551 | 420 | 27 | 24 | [4-45] | [3-91] |
|  |  | 0.25 | 1 | 1 | 1 | 0 | 0 | 87 | 0 | 0 |  |  |  |
|  |  | 0.25 | 2 | 2 | 19 | 1 | 5 | 1425 | 65 | 5 | 80 | [80-80] | [80-80] |
|  |  | 0.25 | 6 | 6 | 1 | 0 | 0 | 90 | 0 | 0 |  |  |  |
|  |  | 0.25 | 7 | 7 | 17 | 0 | 0 | 1350 | 0 | 0 |  |  |  |
|  |  | 0.25 | 8 | 8 | 1 | 0 | 0 | 70 | 0 | 0 |  |  |  |

Abbreviations: IQR, Interquartile range; N, number of patients; PQ, primaquine
