# Supplementary figures and images for: Efficacy of Single-Dose Primaquine With Artemisinin Combination Therapy on Plasmodium falciparum Gametocytes and Transmission: An Individual Patient Meta-Analysis
Source: J Infect Dis. 2020 Aug 11;225(7):1215–26. doi: 10.1093/infdis/jiaa498 (PMC8974839; doi:10.1093/infdis/jiaa498)

**Supplementary Figure 1**. Study Profile.


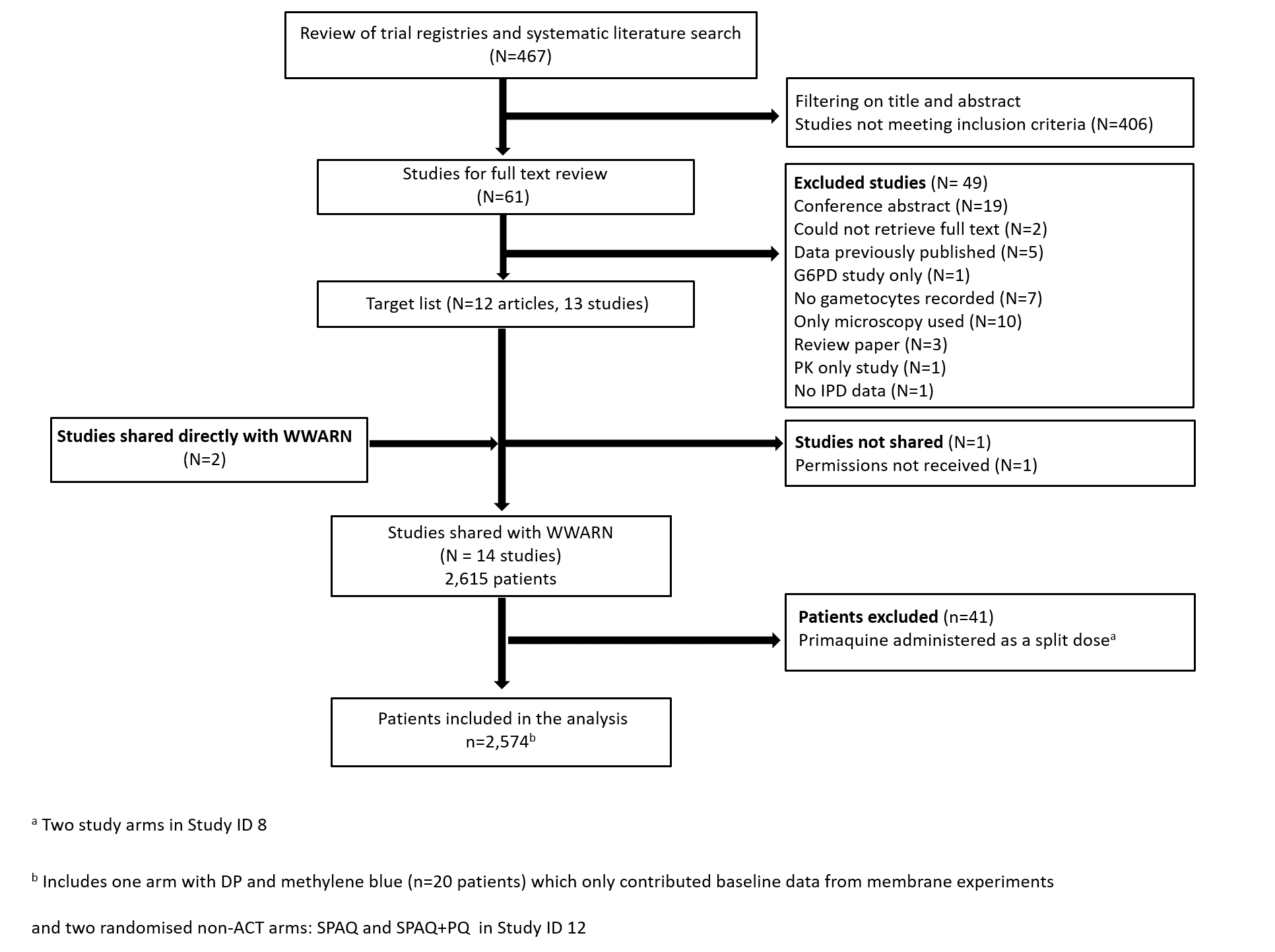

Supplement: jiaa498_suppl_Supplementary_Figure_1 [file jiaa498_suppl_supplementary_figure_1.docx]
