# Supplementary material for: Efficacy of Single-Dose Primaquine With Artemisinin Combination Therapy on Plasmodium falciparum Gametocytes and Transmission: An Individual Patient Meta-Analysis
Source: J Infect Dis. 2020 Aug 11;225(7):1215–26. doi: 10.1093/infdis/jiaa498 (PMC8974839; doi:10.1093/infdis/jiaa498)

**Supplementary Figure 2**. Predicted risk of infecting at least one mosquito in a membrane feeding experiment, after administration of 0.25mg/kg PQ, in relation to gametocyte count at the time of sampling. Results are shown for Day 2 (left panel) and Day 3 (right panel), for treatment with AL (dashed line) or DP (solid line).


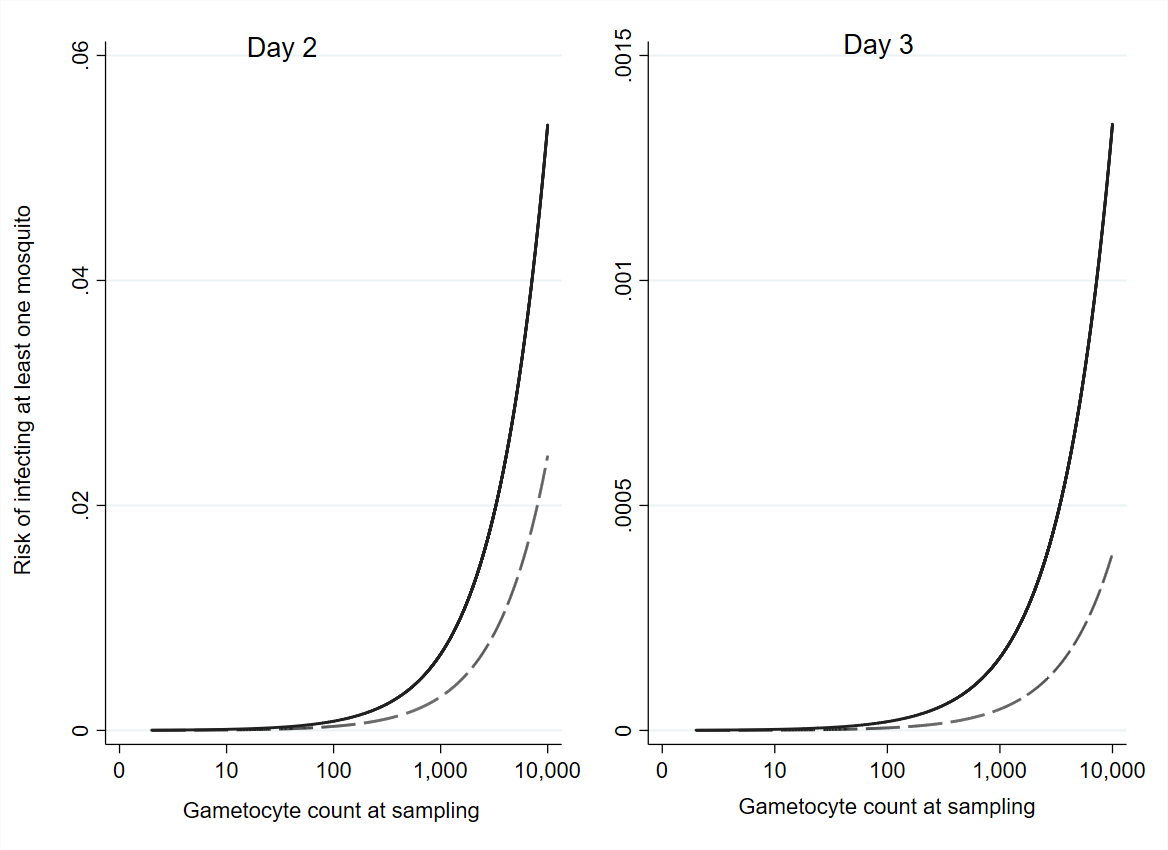

Supplement: jiaa498_suppl_Supplementary_Figure_2 [file jiaa498_suppl_supplementary_figure_2.docx]
