# Supplementary material for: Efficacy of Single-Dose Primaquine With Artemisinin Combination Therapy on Plasmodium falciparum Gametocytes and Transmission: An Individual Patient Meta-Analysis
Source: J Infect Dis. 2020 Aug 11;225(7):1215–26. doi: 10.1093/infdis/jiaa498 (PMC8974839; doi:10.1093/infdis/jiaa498)
Supplement: jiaa498_suppl_Supplementary_Methods [file jiaa498_suppl_supplementary_methods.docx]

**Methods – additional information**

***Systematic Review***

We searched MEDLINE, Web of Science, and Embase, according to the Preferred Reporting Items for Systematic Reviews and Meta-Analyses statement (PROSPERO CRD42019126710, <https://www.crd.york.ac.uk/prospero/display_record.php?ID=CRD42019128185> ). Prospective clinical trials including uncomplicated *P. falciparum* malaria cases or asymptomatic parasite carriers, including patients treated with an ACT and a single dose of PQ (0.75mg/kg or less), published between 1 January 2007 and 30 June 2018, were identified using the following search terms: “(Single OR low) AND dose AND primaquine”.

The identification of eligible studies was conducted by two independent investigators (GSH and IC), with disagreement resolved through discussion. Investigators of targeted studies were contacted and invited to share IPD and any additional unpublished data. Anonymised IPD and supporting documentation of the trials were uploaded into the WorldWide Antimalarial Resistance Network (WWARN) secure repository, and processed according to a data management plan [15]. Study reports generated from the formatted datasets were sent back to investigators for validation or clarification of data shared with WWARN.

***Statistical analysis***

Statistical analyses were carried out using STATA (StataCorp. 2017. Stata Statistical Software: Release 15. College Station, TX: StataCorp LLC.) according to an *a priori* statistical analysis plan [16]. The day on which treatment was first administered was termed day 0. Therefater the prevalence of gametocytaemia on days 7 and 14 was determined seperately for patients with and without gametocytes on enrollment. Univariable and multivariable logistic regression models for gametocyte prevalence, as measured by molecular methods (quantitative reverse-transcriptase-PCR (qRT-PCR) or quantitative nucleid acid sequence based amplification (QT-NASBA)), on each day were fitted with random intercepts for study site. When results from both molecular methods were available, gametocyte density was defined by qRT-PCR. In studies where only gametocyte positivity was determined by a molecular method, density measures by microscopy were included; for samples that were positive by the molecular method but had zero microscopy count, density was assumed to be half of the microscopy detection limit (8 per microlitre, assuming microscopic quantification against 500 white blood cells or 1/16^th^ of a microliter).

The effect of the following baseline covariates was examined: age, gender, log_10_ asexual parasite density, haemoglobin concentration, anaemia (haemoglobin concentration <10g/dL), presence of fever (temp>37.5C) , nutritional status (based on weight-for-age z-scores in children <5 years of age), log_10_ gametocyte density, G6PD status, method of gametocyte measurement (QT-NASBA or qRT-PCR), type of blood schizonticide and dose of PQ administered. Effect of transmission intensity was not explored due to sparse data. **Fractional polynomials** were used to explore nonlinear relationships between continuous covariates and outcome, and these are reported if statistically significant.

Sensitivity analysis was conducted to explore effect of individual studies on the effect of PQ on gametocyte positivity on Day 7 or Day 14. The final models for each outcome were refitted after exclusion of each study in turn and the estimates of the effect of PQ dose were extracted to explore their variability.
